# A Force-Sensing System on Legs for Biomimetic Hexapod Robots Interacting with Unstructured Terrain

**DOI:** 10.3390/s17071514

**Published:** 2017-06-27

**Authors:** He Zhang, Rui Wu, Changle Li, Xizhe Zang, Xuehe Zhang, Hongzhe Jin, Jie Zhao

**Affiliations:** State Key Laboratory of Robotics and System, Harbin Institute of Technology, Harbin 150080, China; zangxizhe@hit.edu.cn (X.Z.); zhangxuehe@hit.edu.cn (X.Z.); hongzhejin@hit.edu.cn (H.J.); jzhao@hit.edu.cn (J.Z.)

**Keywords:** 3-DOF foot-end force sensor, biomimetic hexapod robot, force-sensing system, joint torque sensor, interactive force with terrain

## Abstract

The tiger beetle can maintain its stability by controlling the interaction force between its legs and an unstructured terrain while it runs. The biomimetic hexapod robot mimics a tiger beetle, and a comprehensive force sensing system combined with certain algorithms can provide force information that can help the robot understand the unstructured terrain that it interacts with. This study introduces a complicated leg force sensing system for a hexapod robot that is the same for all six legs. First, the layout and configuration of sensing system are designed according to the structure and sizes of legs. Second, the joint toque sensors, 3-DOF foot-end force sensor and force information processing module are designed, and the force sensor performance parameters are tested by simulations and experiments. Moreover, a force sensing system is implemented within the robot control architecture. Finally, the experimental evaluation of the leg force sensor system on the hexapod robot is discussed and the performance of the leg force sensor system is verified.

## 1. Introduction

**Figure 1:**
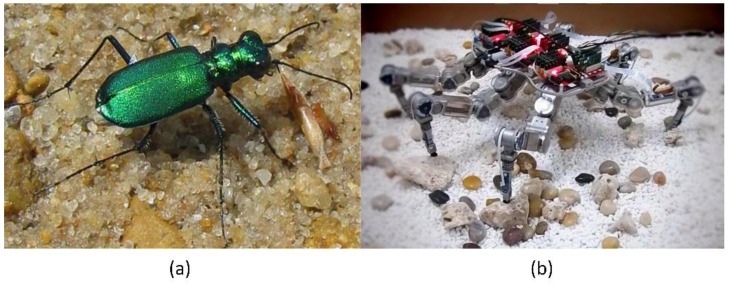
The tiger beetle (**a**) and Biomimetic Hexapod Robot (**b**).

Tiger beetles (Cicindelinae, Figure 1a) have the highest speed of any animal relative to their own body size on land. The distance they can move per second is 171 times longer than their bodies. When running at top speed, a tiger beetle becomes instantaneously blind because of the structural limit of its ommateum and the inadequate processing ability of its brain. However, the tiger beetle can maintain its stability at the highest speed when running on an unstructured terrain, which depends on the physical interaction information between its legs and the ground [1]. This study probes into the biomimetic hexapod robot (Figure 1b), the structure and function of which mimic those of a tiger beetle; this robot attempts to attain independent and stable movements by controlling the interaction force between its legs and an unstructured terrain based on its ability to move rapidly and flexibly [2]. Thus, the force sensing system on the legs of the hexapod robot is remarkably significant to the realization of its stable movement on an unstructured terrain [3].

The force sensing of the legs of a robot is significantly important to improve its walking ability [4,5]. Several creatures with low intelligence, including arthropods, can rapidly respond to certain random events while they walk because some stress responses are triggered by the feedback information from their legs when an external force is applied. The force sensing of the legs is referred as a stress response because this behavior has no prior planning and occurs involuntarily. Creatures consider only two things when they are walking on a plain terrain [6,7], namely comprehensive planning and sector planning. Comprehensive planning, which is similar to the navigation control of mobile robots, selects the appropriate route to a destination; whereas sector planning, which is similar to the path planning of mobile robots, selects the correct path within the vision regardless of the shape of the laying point, stiffness, and ground friction. However, these unknown factors mainly contribute to instability when walking. Most creatures can stabilize their bodies whenever they are running or walking through corresponding adjustments based on the sensing of their legs on interacting force and the estimation of physical gestures. The control of hexapod robots also requires the legs to perceive the interacting force to obtain information about the external environment, thereby independently adjusting their walking strategy. Furthermore, the motion control based on the sensing force information of legs is considerably important to the stability of walking robots due to the high stiffness of their machine bodies [8,9,10].

The strain gauge-type multidimensional force sensor technology provides the basic conditions for the design of the leg force sensing system of a hexapod robot [11]. The elastomer design is particularly important, which determines the sensor structure and performance. Waston and Drake designed a type of multidimensional force elastomer based on the structure of a multiple vertical beam [12,13]. The elastomer in that study had a simple structure and suitable transverse and longitudinal anamorphic effects, but with poor vertical anamorphic and has high coupling problems among different dimensions. In the early 1980s, the Stanford Research Institute developed a hollow barrel-type elastomer [14] with good linearity and repeatability structure but with low stiffness. Stanford University then launched a type of decussation beam elastomer. This structure type was adopted by the popular Scheinman wrist force sensor [15]. The structure has four cantilever beams in the transverse and longitudinal directions, which form a cross structure. The structure also has appropriate symmetry and a self-compensation function when forming a full-bridge circuit. However, it is unsuitable for complex environments because of its poor overload capacity. Sorli also proposed a type of multidimensional force sensor based on the Stewart platform [16,17]. Several shortcomings are still noted despite its advantage in structure design; that is, the structure is unsuitable for certain scenarios that require small sensors due to its complex structure.

In hexapod robot research focused on how to generate the gait [18], most of the studies are focused on structured terrains, flat terrains and mildly rugged terrains [19,20]. Walking on flat and mild rugged terrain also requires that the leg be able to perceptive the collision between foot-end and ground. The state of the leg is determined by the collision perception. Then the leg state information is provided to the leg motion coordination controller to achieve a stable gait. In this motion control mode, since the hexapod robot configuration has a high stability margin and quasi-static motion characteristics, the gait planning for flat terrain is approximately the same as the gait planning for a mildly rugged terrain. In the above case, the leg only needs to perceive foot-to-ground collisions and contact information. Since the unstructured terrain contains both mildly and severely rugged conditions, autonomous and stable walking is a greater challenge for the hexapod robot. The robot design here is suitable for and can adapt to unstructured terrains and improve the stability of complex terrain movements. Because the robot can not only perceive the contact state and the collision between the foot-end and the ground, but also can further perceive which joint causes the collision between the foot-end and ground, it can realize the contact force measurement of the 3-DOF space between the foot-end and the ground, so as to revise the foot trajectory according to the force information, implementing active control of the foot force. Therefore, this paper designs a leg force sensing system to meet the needs of robot movement over unstructured terrains, and the system can meet the structural characteristics and parameters of the miniaturized hexapod robot.

The previous analysis reveals that the design of the strain gauge-type multidimensional force sensor elastomers has continuously improved sensor performance and that the structure design development trend is becoming increasingly diversified. Each structure has its own advantages and disadvantages, which also prove the flexibility of the strain sensor according to different requirements from another perspective. Given the characteristics of autonomous walking motion control over small-sized hexapod robots on unstructured terrains, the leg force sensor should: (a) conform to the characteristics of the leg structure; (b) have a small volume; and (c) be able to suitably measure the interaction force between the legs and complex terrains.

This study broadly attempts to design a force sensing system that can be completely integrated into the leg structure based on the strain-sensing principle according to the structural characteristics of hexapod robots and the demands on the force interactions in complex terrains. The force sensing system can perceive the interaction force between the legs and the external environment that is generated by all movements in the dexterity space. The elastomer and signal processing module integrate into the structural design of the legs without making the size and shape of the mechanism bigger. Moreover, in order to design a force sensing system that is suitable for hexapod robot walking on unstructured terrains, the design index focuses on the following aspects: the system has appropriate force sensing accuracy and range based on the weight and kinetic characteristics of the aforementioned small-sized hexapod robots. It must have good dynamic characteristics and safety protection mechanisms to measure the interactive force on the complex terrain reliably. First, the overall layout and configuration of the sensing system are designed and formulated based on the leg structure and requirements to measure the interaction force when hexapod robots walk. Second, the joint torque, the measurement module of the leg-end force, and the information processing module are designed. The sensor is then demarcated through simulations and experiments to evaluate and test the performance of a single module. Finally, the comprehensive performance and practical application of the leg force sensing system in the climbing and walking experiments of the hexapod robots on unstructured, rugged terrain are assessed and tested.

## 2. Design of Leg Force-Sensing System 

**Figure 2:**
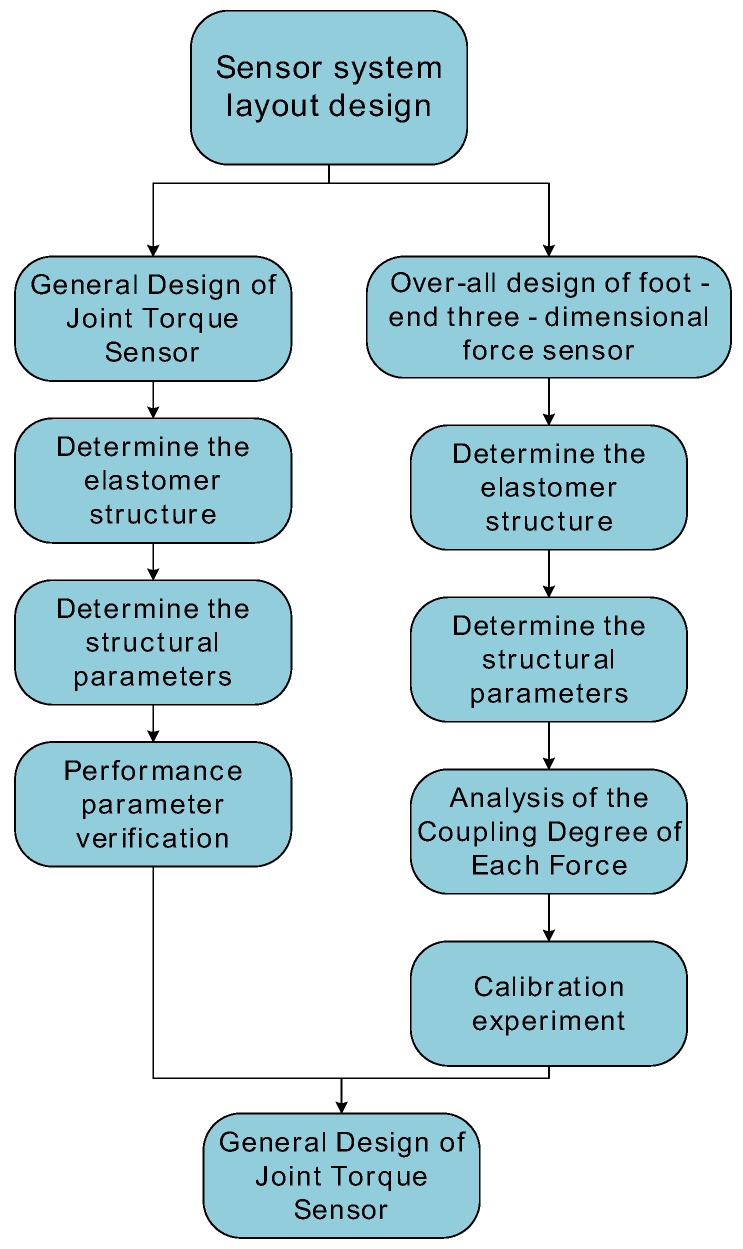
The design process of leg force sensor.

The leg force perception system is based on the strain perception principle, and its design flow is shown in Figure 2. First of all, according to the structure and movement characteristics of the hexapod robot’s leg, the structure of the elastomer and the strain gauge paste location and installation location layout of the information processing module are determined. Then, according to the performance index of the sensor, designed joint torque sensor and foot three-dimensional force sensor respectively. When designing the joint torque sensor, the first thing is to determine the elastic structure and stress measurement location which suit the characteristics of the leg joint structure. Then we select a suitable size and performance parameters for the strain gauge, and the installation methods. Secondly, we design the structure of the elastic body to solve the problem of stress concentration and overload protection. Then, the structural parameters of the elastomer are calculated by the simulation software to obtain the target measurement voltage that matches the signal conditioning circuit. Finally, the performance parameters of the joint torque sensor are evaluated. In the foot-end three-dimensional force sensor design, first, the overall configuration of the three-dimensional force sensor elastomer is determined according to the characteristics of the tibia structure, and the corresponding strain girder of the three-dimensional force measurement is spatially perpendicular to each other, so that the measurement of each dimension is independent and does not interfere with each other. Secondly, according to the principle of stress concentration and overload protection, the overall structure of the three-dimensional force sensor elastomer mounted on the tibia is determined. Then, the structural parameters of the elastomer is determined to make the three-dimensional force to match the three-way signal conditioning circuit. And calculate the degree of coupling between the various forces through the simulation software, to determine whether the interference between the various forces affect the sensor performance indicators. Finally, the performance of the three-dimensional force sensor is evaluated by calibration experiments.

### 2.1. Overall Layout and Performance Parameters of Force-Sensing System

The hexapod robot has quasi-static motion characteristic, which means the basic condition for maintaining stable walking is that at least three non-adjacent legs are in contact with the ground and in supporting phase [21]. The hexapod robot can adopt fixed gaits on flat terrain, and the number of legs in supporting phase and that in the swinging phase is fixed in any time when the robot is walking. The typical gait is three-feet gait [22], but on rugged terrain, the robot needs to adjust the status of each leg in real-time according to the variation of the ground. This kind of gait is named free gait, which judges whether legs are in contact with the ground according to the force-sensing information of legs, and coordinates the statue of each legs based on the designed walking rules.

The biologically inspired hexapod robot design here not only imitates the biological structure of the tiger beetle, but also simulates the motion control method of insects. Insects use nerve reflexes to control movement, and each leg has its own system to judge the leg’s motion status and control the motion of the leg. Only the coordinated movement of the legs is accomplished by the upper level controller, so the legs of the hexapod robot use a modular design, meaning that each leg of the robot has the same structure, the control system and force perception system of each leg are also identical and independent. Each leg’s force information is only provided for its own control module to control the movement of the leg, so that the force information collected by each leg is independent and uncoupling. The parameter design and overall layout of the force sensing system is completely based on the structure, parameters, and motion characteristics of hexapod robots [23], which satisfy the measurement of the interaction force in the walking process on unstructured terrain [24]. The design principles of the force sensing system are as presented as follows:

1) The measurement of the collision perception of the leg during the swinging process with the terrain and its interaction force during the supporting process with the terrain should be accomplished [25,26]. Moreover, it should not only respond to the leg collision in any position when swinging but also completely measure the 3D interaction force between the foot end and terrain during the supporting process. Furthermore, the number of sensors is not redundant.
2) The sensing system is integrated in the leg structure. The sensor elastomer design and signal processing module are consistent with the leg characteristics and do not increase the leg size [27].
3) The sensor must fulfill the required accuracy and measuring range to ensure the interaction force control of the leg. The measurement range depends on the weight and motion type of the robot. Measurement accuracy is determined by the control requirement of actual motion [28].
4) The force sensing system can provide overloaded protection. When robots move on rugged terrain, unexpected collision may cause instantaneous overload and damage the sensor. Thus, designing the protection structure is necessary to prevent overload in the dynamic measurement.

**Figure 3:**
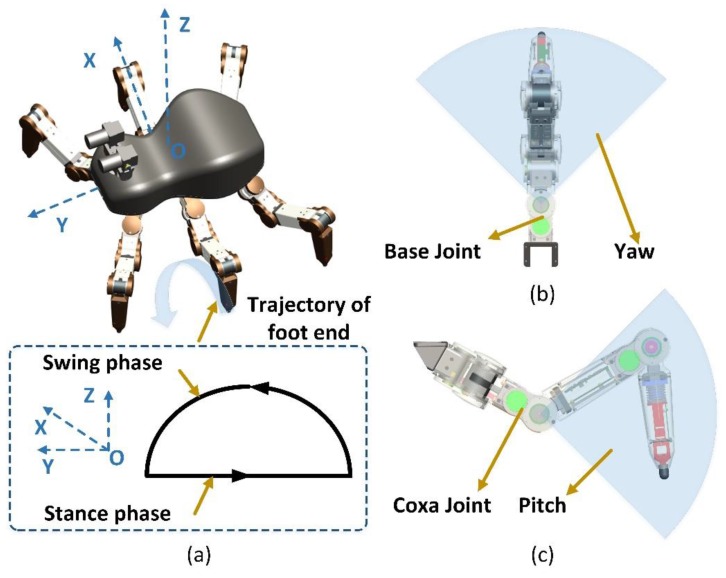
The bionic configuration of entire hexapod robot: (**a**) Different phase of trajectory of foot end; (**b**) The yaw motion of the joint; (**c**) The pitching motion of the joint.

Figure 3a shows the elliptical bionic configuration of the entire hexapod robot. Each robot leg has the same structure and central symmetry in polarization and thus the same force sensing system. Each leg has three degrees-of-freedom (3-DOFs) to realize movement in 3D space. Its foot trajectory is composed of swing and stance phases. When the legs are in the swing phase, the collision between the leg and unstructured terrain is mainly caused by the yaw and the pitching motion of the joint (Figure 3b,c). Therefore, a collision sensing device should be installed in the coxa and femoral joints to perceive any collision in the swing phase [29]. When the legs are in the stance phase, the robot should perceive the 3D interaction force between the foot end and terrain to control the foot-end force. The robot should adjust its pose to improve its walking ability on the unstructured terrain.

**Figure 4:**
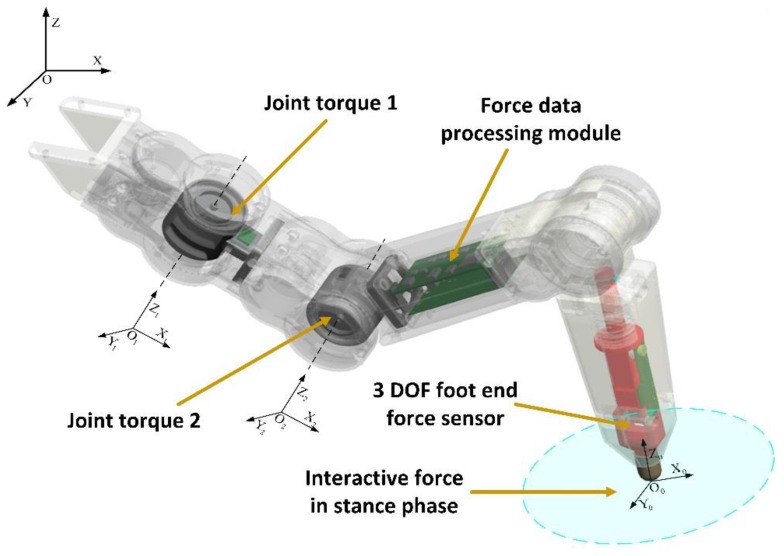
The integrated design of the hexapod robot leg force sensing system.

The overall design of the force sensing system of the hexapod robot leg is shown in Figure 4. This system consists of the coxa joint torque sensor 1, femoral joint torque sensor 2, 3-DOF foot-end force sensor, and force data processing module. The coxa joint torque sensor can detect the collision of parts under the coxa joint caused by the yaw movement of the coxa joint. The femoral joint torque sensor can detect the collision of parts under it, which is caused by the pitch movement of the femoral and tibial joints. Therefore, any collision of legs caused by the joint rotation can be perceived. The sensor obtains the force vector in the joint space, which can also be transformed into the Cartesian space and indirectly measures the interaction force between the foot end and terrain. Nevertheless, the following problems still exist:

1) The singular problem appears when the joint torque transforms to the foot-end force using the Jacobian matrix.
2) Errors are inevitable when the measurement of each joint torque transforms to the force of the foot end. The estimation of the dynamic model can be utilized to compensate the measurement error of joint torque information which always contains other torque forces, such as gravity, inertia forces, Coriolis force, centrifugal force, and friction. However, the method has several natural disadvantages due to the error of the Jacobian matrix caused by the inevitable dynamic model and mechanical errors.

Given the aforementioned reasons, the 3-DOF force sensor is directly placed in the tibia of the leg to measure the foot-end force directly, thereby improving the measurement accuracy and lowering the amount of data processing. Furthermore, the 3-DOF foot-end force sensors not only perceive the force vector in the foot coordinate but can also obtain the force vector in the Cartesian space through the Jacobian matrix.

The force information processing module is set within the femur, which can fully employ the inside space of the femur and decrease the line distance between the multiple sensors and processing module. However, the limited space restricts the module size and configuration.

The range of the joint torque sensor is generally consistent with the joint output torque [30]. The maximum operating torque in this study is 1.9 Nm, and the distance between the joint output shaft and the strain cantilever of the elastic body is 25 mm. Thus, the design range of the joint torque sensor is 2 Nm with a discrimination of 2 Nm, which refers to the safety collision force of the robot design criteria. The total weight of the robot is 3.6 kg, at least three adjacent legs support its body when walking, and its walk can be characterized by quasi-static movements; given these factors, the ranges of the 3-DOF foot-end force sensors are set at 30 N in the normal direction $F_{Z}$, tangential direction $F_{X}$, and transverse direction $F_{Y}$. The discrimination is set at 0.03 N according to the safety collision force. The configuration and structural parameters are constrained by the leg structures. The specific parameters are listed in Table 1.

### 2.2. Joint Torque Sensor

#### 2.2.1. Conceptual Design of Joint Torque Sensor

**Figure 5:**
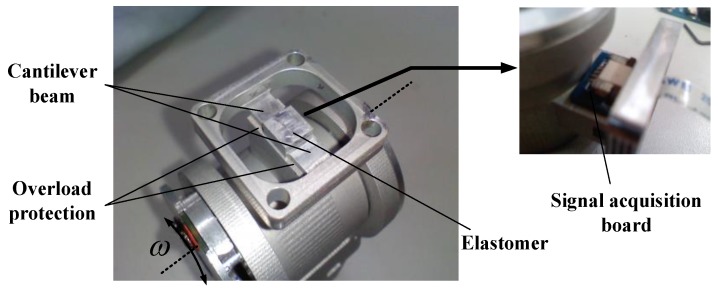
Picture of the joint torque sensor.

The designed joint torque sensor is shown in Figure 5 [31]. The *k* of the sensor is made of duralumin and integrated in the flange that connects the joint output stage to the next limb. Hence, it can directly perceive the torque of the joint output and create a good linear relationship between the elastic strain and joint torque. The elastomer of the sensor utilizes the cantilever beam structure of the double straight arm, and the radial direction of the beam is consistent with the rotation direction of the joint. According to the principles of concentrating stress, the structure is relatively weak when compared with the fixed structure on both ends. The axial direction of the cantilever beam is perpendicular to the output direction of the joint torque, which undermines the effect on the overall stiffness of the leg due to unavoidable weakness design. Given that the overload will damage the sensor and even the robot structure, the overload protection function is considered in the structural design of the elastomer. 

**Figure 6:**
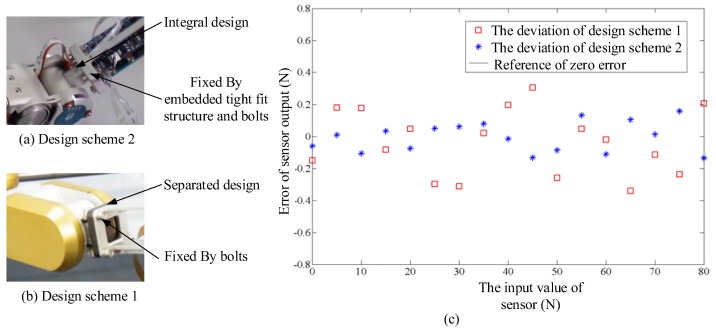
Comparison of two design schemes: (**a**) Scheme after improved; (**b**) Original scheme; (**c**) Comparison of actual measurement error

Figure 6 shows that the fixed platform is longer than the linking site of the beam and platform. When the beam experiences serious deformation caused by overloads, the convex structure of the fixed platform can support the beam, thereby eliminating the effect of the cantilever beam and enhancing the strength of the entire elastomer. The signal acquisition board of the sensor is fixed on the elastomer according to the principle of proximity. The signal processing module will input voltage to the strain gauge and collect the output voltage of both bridge ends through the FPC line. Figure 6a shows that the elastomer of the joint torque sensor and joint output stage adopt an integrated design in design schemes 2 (Figure 6b) and 1 through the analysis of scheme 1. The flange and next leg section are joined by bolts and a specially designed, embedded, tightly-fit structure for the flange to enhance the stability of the two fixed ends, thereby improving the measurement performance of the sensor. Figure 6c shows the errors in the actual measurement after calibrating both sensors in design schemes 1 and 2. Theoretical linearity can be expressed as follows:

(1) $$\delta_{L}=\frac{\Delta_{max}}{Q_{n}}\cdot 100\%$$

where $\Delta_{max}$ denotes the maximum deviation of the actual measured value after the smooth fitting, and $Q_{n}$ is the theoretical maximum upper limit.

The calculated results show that the sensor linearity values in design schemes 1 and 2 are 0.37 FS% and 0.16 FS%, respectively. Therefore, design scheme 2 significantly improves the sensor linearity compared with the initial design scheme 1, in which the elastomer is a separate component connected to the joint and lower limb by bolts (Figure 6a,b).

#### 2.2.2. Parameter Design of Joint Torque Sensor

**Figure 7:**
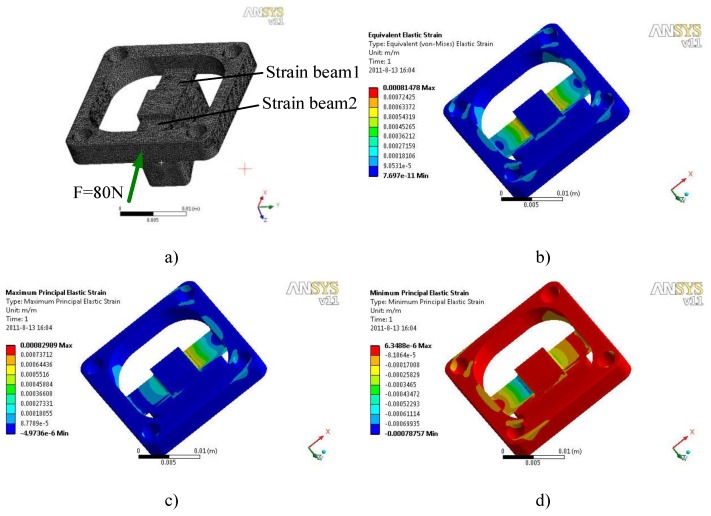
Finite element simulation of joint torque sensor elastomer: (**a**) Finite element model; (**b**) Integrated strain; (**c**) Maximum positive strain; (**d**) Maximum negative strain.

For the strain gauge force sensor [32], the elastomer is an important factor that determines the sensor performance. Therefore, the location for pasting the strain gauge and reasonable structural elastomer parameters are obtained through the finite element analysis based on the sensor design target and strain gauge parameters. The maximum working torque of the joint is 1.9 Nm, and the maximum output force of the flange along the joint torque output direction is 76 N. Figure 7 shows the observation of the strain beam deformation under a full scale when exerting an 80-N force along the direction of the joint torque output upon the flange. 

Figure 7a shows that the elastomer strain is concentrated in the cantilever beam, which is expected when exerting force upon the flange. A closer distance from the connection indicates a larger strain. Therefore, the strain gauge end was pasted, which places the resistance wire along the connection edge, thereby improving the sensitivity level of the sensor and ensuring the accuracy of the paste orientation of the strain gauge. Figure 6c and Figure 7b show the maximum and minimum (reverse maximum) strains that are concentrated in the upper surfaces of beams 1 and 2 when the force is applied, respectively. The strain gauge of the sensor adopts the J2A-06-S185N-10C micro-strain gauge of Vishay Micro-Measurements (Raleigh, NC, USA). The strain gauge is a double-line type, the backing of which integrates two groups of sensitive grids that are equivalent to two strain gauges. The base area of the strain gauge has a small area (6.1-mm length and 6.4-mm width), thereby reducing the area of the strain beam. The resistance of the strain gauge is relatively high at 1000 R, which can withstand a large voltage with high sensitivity (the nominal value of the sensitivity coefficient *K* = 2.05), low power consumption, and small temperature drift.

Figure 7 shows that the strain gauge is pasted symmetrically on the upper surface of the two strain beams in the elastomer. Four resistors are used in strain gauges 1 and 2. These resistors build a full-bridge circuit for voltage acquisition, which is connected by a signal acquisition board, and connect the input and output circuits of the voltage with the signal processing module. Relative to the half-bridge circuit, the full-bridge circuit can effectively inhibit the output voltage drift of the bridge, which is caused by temperature and creep. The optimal bridge voltage can be expressed as by the following empirical formula:

(2) $$U_{0}=2\sqrt{RP_{g}^{\prime }F_{g}}$$

where the optimal bridge voltage $U_{0}$ is modeled as a function of the strain gauge resistance R(Ω), area of the sensitive grid $F_{g}\left(m^{2}\right)$, and the power density of the sensitive grid $P_{g}^{\prime }\left(W/m^{2}\right)$.

The power density is set at $P_{g}^{\prime }=1.6\times 10^{-3}\left(W/m^{2}\right)$; thus, the calculation of the optimal bridge voltage is 5.6 V, and the power supply voltage of the information processing module to the data collection board is set at 5 V. The output voltage of the full-bridge circuit is expressed as follows:

(3) $$U_{out}=\frac{K\cdot \left(\varepsilon_{1}-\varepsilon_{2}+\varepsilon_{3}-\varepsilon_{4}\right)}{\left[2+K\cdot \left(\varepsilon_{1}+\varepsilon_{4}\right)\right]\cdot \left[2+K\cdot \left(\varepsilon_{2}+\varepsilon_{3}\right)\right]}\cdot U_{in}\;\approx \frac{1}{4}\cdot K\cdot \left(\varepsilon_{1}-\varepsilon_{2}+\varepsilon_{3}-\varepsilon_{4}\right)\cdot U_{in}$$

where K is the sensitivity coefficient, and $\varepsilon_{i}$ is the magnitude of the strain gauges that correspond to resistance 1.

The strain gauge is the same, and the distribution is symmetrical. Therefore, $\varepsilon_{1}=-\varepsilon_{2}=\varepsilon_{3}=-\varepsilon_{4}=\varepsilon$. Equations (3) and (4) yield:

(4) $$U_{out}=K\cdot \varepsilon \cdot U_{in}$$

The terminal reference voltage for AD conversion of the signal processing module is 5 V, the reference voltage of the amplifier is 2.5 V, and the amplification multiple is 199.4. Therefore, the input voltage should not exceed $U_{out}=\pm 1.25\times 10^{-2}\;V$. From Equation (4), the full variation range of the dependent variable $\varepsilon =\pm 1.2\times 10^{-3}$ mm can be obtained. The finite element calculation results in Figure 7 reveal that the position of the full-scale strain value of the patch conforms with the design requirements when the strain beam is 8-mm long, 6-mm wide, and 2-mm thick. Furthermore, the distance between the beam of the overload protection structure and platform is 0.3 mm. The finite element calculation determines that the security coefficient of the beam under full scale is 4.32. Thus, when the safety factor is decreased to 2.28 by increasing the force on the beam, the overload protection function is produced, the overall strength of the elastomer is instantaneously improved, and the appropriate strength and high sensitivity of the elastomer is ensured simultaneously. The performance parameters of the joint torque sensor are listed in Table 2. 

### 2.3. Development of Foot-End 3-DOF Force Sensor

#### 2.3.1. Configuration of Elastomer

**Figure 8:**
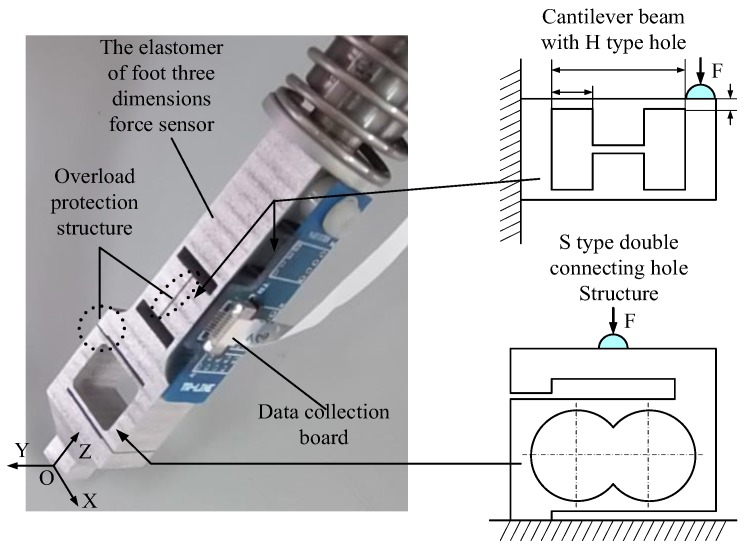
Foot 3-DOF force sensor and elastomer strain principle.

Figure 8 shows that the structures of the 3D foot-end force sensor and its elastomer have the following characteristics:

1. *The strain beam along the radial distribution of the tibia*: The design goal of the leg force sensing system entirely combines the leg structural characteristics. The structural characteristics of the tibia include a long radial length and relatively small dimension, which lowers the structural constraint and improves leg flexibility. The strain gauge should not be small for the area of the strain gauge that directly determines the elastomer size to ensure the sensor performance. Hence, the elastomer shape and volume can be effectively improved by properly configuring the distribution of the elastic strain structure. At present, the majority of elastic strain beam is in plane distribution. For example, the crossed beam and structural strain beam are in the horizontal arrangement along the outer rim. This type of structure is suitable for the multi-axis force sensor of the wrist, which has a large area. The distribution design of the strain structure along the radial direction for thin legs should be developed.
2. *The coupling of different dimensions is small*: For a better performance of the strain properties in the normal, transverse, and tangential directions, the existence of the coupling among different dimensions cannot be avoided whether in the vertical beam structure or crossed-beam structure due to the selection of the configuration and structure parameters. Decreasing or even eliminating the coupling among the different dimensions from the perspective of the elastomer can avoid system errors caused by the decoupling operation. Particularly, when the sensor is applied to the complex environment, the characteristic matrix based on the static decoupling algorithm is unsuitable for the dynamic measurement of the emergency, which may increase the transmission error.
3. *Overload protection design*: Walking in a complex environment imposes a high demand on the ability of the sensor to adjust to unexpected scenarios. The overload protection structure can effectively improve the reliability while ensuring the sensitivity level of the sensor.
4. *Integrated design*: A multi-axis force sensor elastomer has a complex structure, which is more difficult than a single-machined component. Therefore, the elastomer design should be easily machined as soon as possible to attain the design requirements of integration.

**Figure 9:**
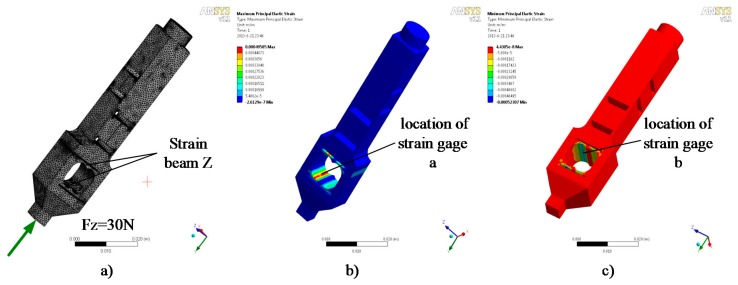
Analysis of the normal stress of the strain beam: (**a**) Finite element model; (**b**) Maximum positive strain; (**c**) Maximum negative strain.

As shown in Figure 8, the 3D foot-end force sensor integrated in the tibia structure is composed of an elastic body and information acquisition board. Each dimensional induction corresponds to the strain structure separately. The elastomer beam perceives the normal force using an S-shaped double-hole structure, which has the advantages of high sensitivity in the sensitive direction, concentrated strain value, high stiffness in the non-sensitive direction, and small transverse interference. This structure is suitable for measuring the force in the vertical direction. The applicable range of force measurement is $10\;N\;\mathrm{to}\;10^{3}\;N$. Figure 9 shows that when the normal force is applied to the finite element model, the strain is concentrated in the sensitive part of the normal direction. Given the placements of the patch positions a and b in Figure 9, the maximum positive and negative strain values are produced in the central axes of the upper and lower surfaces in the same hole. 

**Figure 10:**
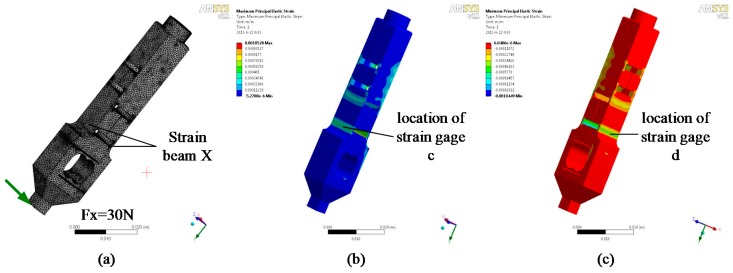
Analysis of the transverse stress of the strain beam: (**a**) Finite element model; (**b**) Maximum positive strain; (**c**) Maximum negative strain.

**Figure 11:**
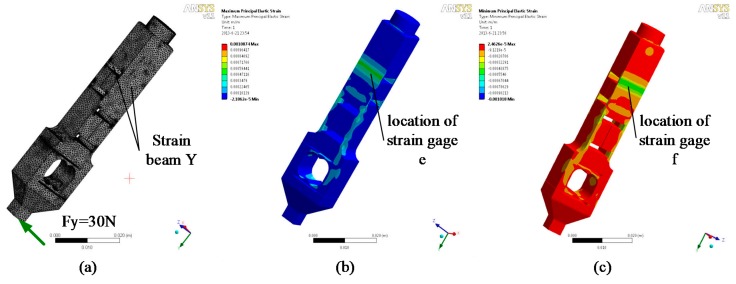
Analysis of the tangential stress of the strain beam: (**a**) Finite element model; (**b**) Maximum positive strain; (**c**) Maximum negative strain.

The H-shaped-hole cantilever beam structure is adopted for the strain beam, which has transverse and tangential forces. This type of elastomer structure has the advantages of appropriate rigidity, high sensitivity, and excellent stability. As shown in Figure 10 and Figure 11, the transverse and tangential forces are applied to the elastomer finite element model as strain positions c, d, e, and f. The positive and negative maximum strains occur at the central lines of the upper and lower surfaces of the sensitive part of the strain beam.

#### 2.3.2. Analysis of the Effect of Dimensional Coupling on Sensor Performance

The strain gauge-type force sensor perceives the external force through the elastomer strain. The strain value can be converted to a measurable signal by the strain gauge, which outputs the voltage U. The relationship between voltage U and loading force F can be expressed as follows:

(5) U=C·F

where C is the eigenmatrix value of the multi-axis force sensor, which reflects the elastomer characteristics, such as the coupling extent and amplification multiple of the signal.

The errors ΔU and ΔF always exists in the measurement of voltage U, carrying force F, and ΔC. The eigenmatrix error has a decisive impact on the system. Thus, the actual mathematical model of the sensor is expressed as:

(6) U+ΔU=(C+ΔC)·(F+ΔF)

Given the genetic formula of the error and matrix perturbation theory [33,34], the propagation of error of loading force F during the transformation can be expressed as follows:

(7) $$\varepsilon_{F}=\left(\varepsilon_{U}+\varepsilon_{C}\right)\cdot k\left(C\right)$$

where $\varepsilon_{F}=∥\Delta F∥/∥F∥$, $\varepsilon_{U}=∥\Delta U∥/∥U∥$, and $\varepsilon_{C}=∥\Delta C∥/∥C∥$ are the relative error of the sensing system, the relative error of the bridge output measurement, and the acquisition error of the eigenmatrix, respectively. k(C) is the factor of error propagation, which is mainly determined by the condition number cond(C) of the eigenmatrix C, and $cond\left(C\right)=∥C∥\cdot ∥C^{-1}∥$. Thus, the system error of the sensor is mainly determined by the transmission error of the eigenmatrix C. Most of the eigenmatrix C is calculated based on static calibration method; however, the dynamic responses of the elastomer in different sensitive directions are always different due to its asymmetric structure. Therefore, introducing transmission errors in the dynamic measurement is inevitable. Hence, if the elastomer design can lower the coupling among different dimensions based on satisfying the measurement requirements, then sensor performance and stability will be largely improved. Any 2D force beam on this elastomer is independent and perpendicular to each other; thus, no dimensional coupling occurs in the structure. However, when each dimensional force works independently, it will also affect the sensitive structure in other directions because of the integrated design of the elastomer structure. As shown in Figure 9, Figure 10 and Figure 11, the finite element simulation results reveal that when the maximum force is placed in a single direction of the foot end, the strain of the corresponding direction increases, but the strains of other sensitive directions and patch location of the strain gauge become extremely small.

#### 2.3.3. Structural Parameter Design of Sensor

The elastomer material is LY 12 Duralumin [35], with elastic modulus $E=0.72\times 10^{6}\;\mathrm{kg}/{\mathrm{cm}}^{2}$, Poisson’s ratio μ=0.33, and density $\rho =2.7787\times 10^{3}\;\mathrm{kg}/m^{3}$. The 3D force sensor also uses the Vishay Micro-Measurements model number J2A-06-S185N-10C double-straight type strain gauge [36], and the supply voltage is 5 V to ensure that the signal processing and the power supply circuit of the leg force sensing system are consistent. The reference voltage of the AD conversion on the digital signal processing module terminal is 5 V, the reference voltage of the amplifier is 2.5 V, and the magnification is 199.4. The expected full range of the strain ε is $\left[-1.2\times 10^{-3},1.2\times 10^{-3}\right]\;\mathrm{mm}$ using Equation (4). 

The S-shaped double-hole structure can be simplified into a two-degree statically indeterminate beam, with one end of the beam fixed and the other performing a translational motion along the force-sensitive direction:

(8) $$\sigma =\frac{M}{W},\varepsilon =\frac{\sigma }{E},W=\frac{bh^{2}}{6}$$

where σ represents the bending stress, ε denotes strain, W is the section modulus in the torsion of the shaft, M is the bending moment, E is the modulus of elasticity, b is the beam width, and h is the beam thickness. Given the double-beam structure, the force experienced by each beam is F/2. Thus, the strain at the sensitive location of the beam is:

(9) $$\varepsilon =\frac{3F\left(l-\frac{\alpha }{2}-\delta \right)}{bh^{2}E}$$

where F is the loading force, l represents the length from the beam end to the geometric center, α is the length of the strain gauge backing, and δ is the distance from the end of the beam to the edge of the strain gauge.

From Equation (9), after the type and sticking position of the strain gauge are determined, the strain of the S-shaped double-hole elastomer only relates with the width and thickness of the beam and the length from the beam end to the geometric center. According to the overall structure of the legs and the size of the strain gauge, the width of the beam b=5 mm and the length from the beam end to geometric center l=5 mm. Therefore, the thinnest beam thickness h=1.2 mm can be calculated using finite element method according to the expected strain. The distance between the beam and the platform of the overload protection structure is 0.2 mm, and the security coefficient of the beam under full scale is 3.96 on account of the analysis. When the safety factor is decreased to 2.66 due to the increasing force on the beam, the beam and the platform of the overload protection structure come into contact, thereby improving the strength of the elastomer in this direction.

Like the S-shaped double-hole structure, the H-type cantilever beam structure perceiving transverse and tangential forces can also be simplified to a two-degree statically indeterminate beam, the sensitivity of which is closely related to the size of its H-shaped hole aside from the structural beam parameters. According to the length of the sensitive grid of the strain gauge, the width of the H-shaped hole can be determined ($l_{1}=$ 2.2 mm). The entire structural beam parameter can be determined by the limitation of the length of the leg (total length $l_{3}=$ 12 mm, width $d_{1}=$ 10 mm). The beam thickness ($d_{2}=1.1\;\mathrm{mm}$) is calculated using finite element method according to the expected strain. The length of overloading protection structure $l_{2}=$ 7.6 mm, and the distance between platforms is 0.2 mm. When full-range force acts on the sensitive direction of the beam, the safety factor of the transverse and tangential strain beams are 4.56 and 4.23, respectively. By continuously increasing the loading force until the platforms of the overload protection structure contact with each other, the safety factor is decreased to 1.96 and 1.95, which prevents exceeding the yield strength of the beam due to overload. The natural frequencies of the elastomer under various vibration modes can be measured through the modal analysis of the finite element model of the elastomer. Therefore, the work bandwidth of the 3D force sensor is 0–1792 Hz. The results are shown in Table 3.

Therefore, the work bandwidth of the 3-DOF force sensor is 0~1792 Hz.

#### 2.3.4. Simulation Analysis of Elastomer’s Coupling Degree

A single direction and homogeneous change force are loaded on the finite element model, and the dependent variable of the strain gauge on each sensitive direction of the strain beam is then recorded. The coupling degree of the elastomer can be obtained as follows:

(10) $$\Delta_{ijk}=\frac{\varepsilon_{ijk}}{\varepsilon_{ifs}}\times 100\%$$

where $\Delta_{ijk}$ is the coupling rate of the measuring point k on the sensitive direction i under the loading force direction j. $\varepsilon_{ijk}$ is the beam strain in the patch location of the strain gauge on the measuring point k along the sensitive direction i, and the force is loaded on the direction j. $\varepsilon_{ifs}$ is the beam strain in the patch location of the strain gauge on the sensitive direction i, where the force is in full range. Thus, the coupling degree of sensitive direction i when force is loaded on the direction j can be expressed as follows:

(11) $$D_{ij}=\sqrt{\frac{1}{n-1}\cdot \overset{n}{\underset{k=1}{\sum }}\Delta_{ijk}^{2}}\times 100\%$$

**Figure 12:**
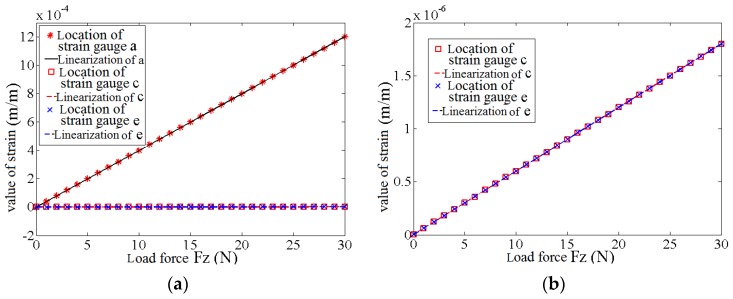
The strain of strain gauge’s patch location on the elastomer under the loading force $F_{Z}$: (**a**) The strain on all directions; (**b**) The strain on non-sensitive directions.

Force is loaded on the finite element model of the elastomer, ranging from 0 N to 30 N with a step length of 1 N. The changing curve of the beam strain in the patch location of the strain gauge on various sensitive directions can be obtained, as shown in Figure 12. Figure 12a shows the comparison of the strains in patch locations a, c, and e of the strain gauge; whereas Figure 12b shows the comparison of the strains in patch locations c and e of the strain gauge. Under the action of the loading force $F_{z}$, the strain in the patch location a of the strain gauge in the Z-direction is sensitive and has low nonlinearity. However, strains in patch locations c and e of the strain gauges in the X- and Y-directions are minimal. According to Equation (11), under the loading force $F_{z}$, the coupling degree in the patch location of the strain gauge of strain beam c in the X-direction is 0.005%, which is similar to that in the patch location of the strain gauge of strain beam e in the Y-direction.

**Figure 13:**
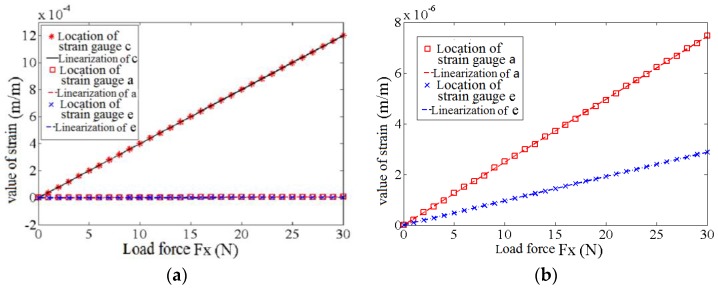
The strain of strain gauge’s patch location on the elastomer under the loading force $F_{X}$: (**a**) The strain on all directions; (**b**) The strain on non-sensitive directions.

Force $F_{x}$ is loaded on the finite element model of the elastomer using the same method. Figure 13a shows the comparison of the strain in patch locations a, c and e of the strain gauge, whereas Figure 13b shows the comparison of the strain in patch locations a and e of the strain gauge. Under the action of loading force $F_{x}$, the strain in patch location c of the strain gauge in the X-direction is sensitive and has low nonlinearity, whereas the strains in patch locations a and e of the strain gauges in the Z- and Y-directions are minimal. According to Equation (11), under the loading force $F_{x}$, the coupling degree in the patch location of the strain gauge of strain beam c in the Z-direction Z is 0.021%, whereas that in the patch location of the strain gauge of strain beam e in the Y-direction Y is 0.008%.

**Figure 14:**
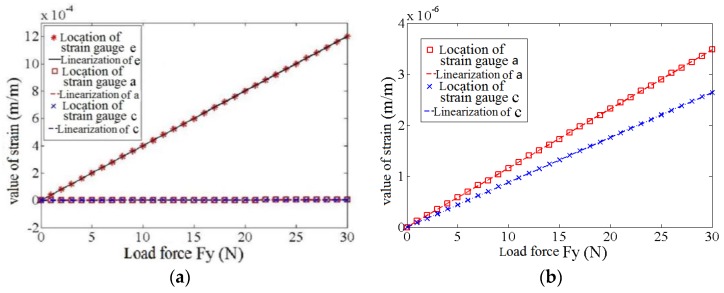
The strain of strain gauge’s patch location on the elastomer under the loading force $F_{Y}$: (**a**) The strain on all directions; (**b**) The strain on non-sensitive directions.

Force $F_{Y}$ is loaded on the finite element model of the elastomer using the same method. Figure 14a shows the comparison of the strains in patch locations a, c, and e of the strain gauge, whereas Figure 14b shows the comparison of the strains in patch locations a and c of the strain gauge. Under the action of the loading force $F_{y}$, the strain in patch location e of the strain gauge in the Y-direction is sensitive and has low nonlinearity, whereas the strains in patch locations a and c of the strain gauges in the Z- and X-directions are minimal. According to Equation (11), under the loading force $F_{Y}$, the coupling degree in the patch location of the strain gauge of strain beam e in the Z-direction Z is 0.010%, whereas that in the patch location of the strain gauge of strain beam e in the X-direction X is 0.007%.

The simulation result shows that the coupling degree of the elastomer between the different dimensions of the 3D force sensor is minimal. The AD conversion interface of the information processing module shows that the maximum discrimination of the sensor is 0.1%. Therefore, the elastomer can nearly ignore the coupling effect among different dimensions.

#### 2.3.5. Calibration Experiments for the 3-DOF Force Sensor 

**Figure 15:**
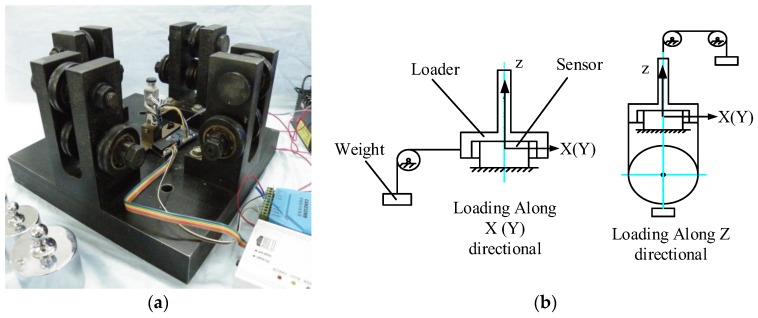
Calibration of 3-DOF force sensor: (**a**) The calibration platform of the calibration experiment; (**b**) The calibration principle of the calibration experiment.

**Figure 16:**
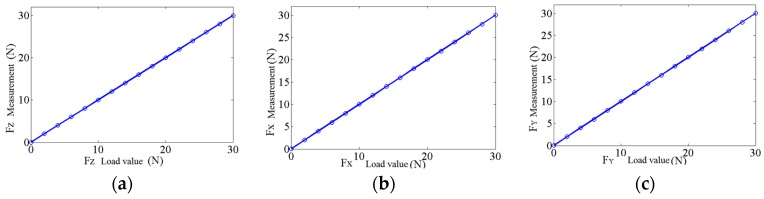
Error analysis of 3-DOF force sensor: (**a**) The measurement of Z axis; (**b**) The measurement of X axis; (**c**) The measurement of Y axis.

Sensor calibration is used to determine the static performance index of the sensor. Given its complex structure and multiple interference factors, the multi-axis force sensor must be calibrated to improve its performance. The weight hammer method is used to calibrate the foot end 3-DOF-force sensor. The calibration platform and principle are as follows (Figure 15): 

A fixing frame is used to pitch the sensor into the center of the calibration platform. The weight is loaded onto the sensor through the pulley and the loader (the accuracy of the weight is 0.05%). The load from the initial state is increased to the full range with 200 g as the interval along the positive and negative directions of the X-, Y-, and Z-axes. The load is then gradually reduced to zero during the same interval. This process is repeated six times, and the measurement data are recorded. Finally, the calibration result of the sensor is obtained, as shown in Figure 16. The performance parameters of the sensor can also be obtained based on the result.

To evaluate the precision index of the sensor, its error should be analyzed. The error of the multi-axis force sensor can be evaluated using Type I and Type II methods. 

Type I is the deviation between the measured and actual values in the loading direction. This method reflects the composite error of the sensor under the single-loading direction, which can be expressed as follows:

(12) $$\delta_{i}=\sqrt{\frac{1}{n-1}\cdot \overset{n}{\underset{k=1}{\sum }}\delta_{ik}^{2}}\times 100\%,$$

where:

(13) $$\delta_{ik}=\frac{F_{mik}-F_{rik}}{F_{ifs}},$$

where $\delta_{ik}$ is the error of the measuring point k on the loading direction i, $F_{mik}$ is the measurement of the measuring point k on the loading direction i, and $F_{rik}$ is the actual value of the measuring point k on the loading direction i.

Type II is the deviation between the measured and actual values of the other sensitive directions caused by the force acting in the loading direction. This method reflects the errors of the multi-axis force sensor caused by the coupling between different dimensions and can be expressed as follows:

(14) $$D_{j}=\sqrt{\frac{1}{n-1}\cdot \overset{n}{\underset{j=1,j\neq i}{\sum }}D_{ij}^{2}}\times 100\%.$$

The error of the 3D foot-end force sensor in all loading directions can be obtained using Equations (12) and (13). The result is shown in Table 4.

Table 4 shows that the Type II method is two orders of magnitude smaller than Type I in numerical value. Therefore, the coupling effect among different dimensions on the measurement accuracy can be ignored and eliminating it through decoupling calculation is unnecessary, thereby avoiding the introduction of system error through the transfer matrix and improving measurement precision and stability. 

The performance parameters of the 3D foot-end force sensor on account of the calibration data are shown in Table 5. The measuring range of the 3D foot-end force sensor is dependent on the driving force limit of the robot, and the discrimination should meet the requirement of the force control. Furthermore, the sensor should have a low degree of nonlinearity, hysteresis, and repeatability, as well as ensure measurement accuracy. The wide bandwidth ensures the stability of dynamic measurement. The above performances of the sensor meet the requirements of real-time measurement of foot-end force that is applied on rough terrain walking.

## 3. Design of the Force Information Processing Module

**Figure 17:**
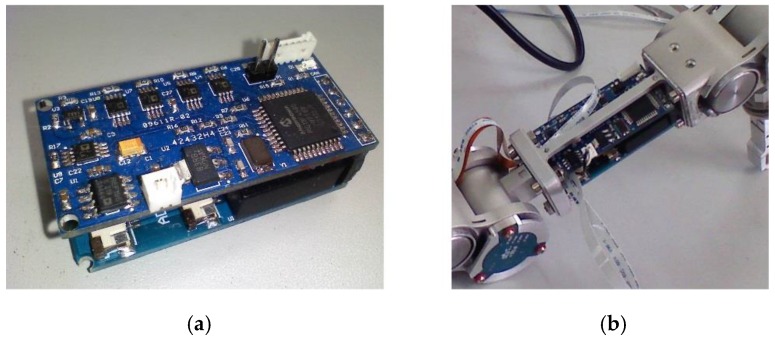
Force information processing module: (**a**) PCB of module; (**b**) Module is assembled in the coxa of leg.

As mentioned earlier, the hexapod robot legs have a modular design, whereby each leg has a separate force information processing module. As shown in Figure 17, the force information processing module of each leg integrates the force information acquisition and processing functions of the two joint torque sensors and the foot end 3-DOF force sensor. Since the measured forces are not coupled, each measurement of the force information corresponds to a bridge made up by the strain gauge. The 3-DOF force measured by the 3-DOF force sensor also corresponds to a bridge and according to the output voltage of the respective bridge we measure the strain of the elastic body to get the value of the force. Therefore, the leg force perception system needs a separate amplifier circuit to collect the five force signals, meaning the perception system needs independent collection and processing capabilities for the five force signals.

The force information processing module is shown in Figure 17. This module is integrated into the thigh of the legs, which is located in the middle of each force sensor; thus, collecting and transporting information is easy. The force signal acquisition board of each sensor is composed of a full-bridge circuit by the PCB internal circuit, and a 5-V bridge-end input voltage is provided by the force information processing module. Five output voltage signals are transmitted to the force signal processing module through the FPC line, and the signal is transmitted to a single-leg controller via CAN communication after being processed.

**Figure 18:**
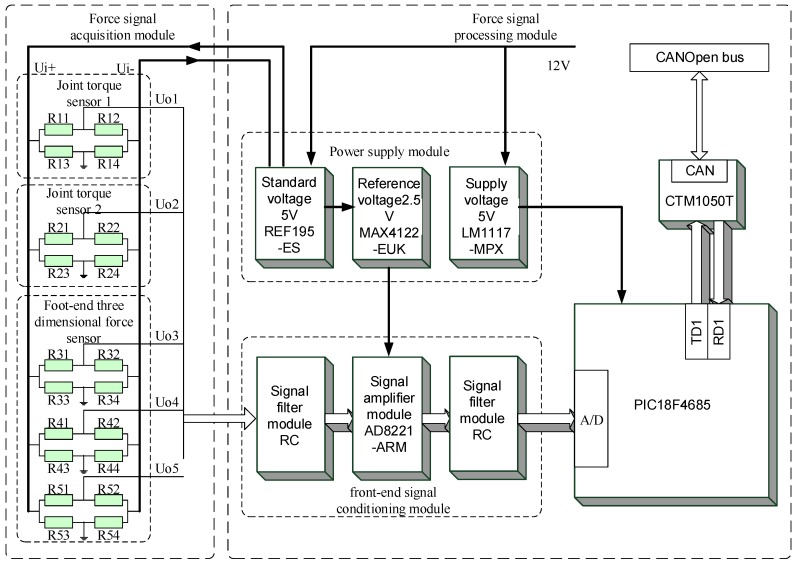
Schematic diagram of force information acquisition and processing.

Figure 18 shows that the force signal processing module uses the PIC18F4685 microcontroller by MICROCHIP (Chandler, AZ, USA) as the core, which is supplemented by the front-end signal conditioning module and power supply module. This chip uses ECAN^TM^ and nanoWatt technology and can support CAN communication effectively with low power consumption. The chip also integrates 11 channels of 10-bit A/D conversion modules, thereby obtaining multichannel force information accurately. In the information processing module, this chip obtains the power needed through the LM1117 chip of the power supply module, which can transform the external voltage (12 V) to a supply voltage (5 V). The integrated CTM1050TCAN transceiver module is used to convert the logic level of the CAN controller to the differential level; thus, one node can communicate with the other nodes of the CAN bus.

The processing module uses the REF195-ES chip to produce the standard voltage (5 V), thereby ensuring the accuracy and stability of the bridge input voltage. This chip has the advantages of high precision and low temperature drift, and its maximum error is 2 mV. The temperature coefficient is 5 ppm/C, and the maximum output current is 30 mA. The output voltage of the bridge collected by the power signal acquisition module is very weak and has clutter, thus, it must be filtered and amplified. First, the front-end signal conditioning module uses the RC circuit to filter the received signal and then uses an AD8221-ARM chip to amplify the signal. The chip has good DC performance features, such as low noise and high precision, and it uses MSOP package to save space. It also has a wide range of gain to be selected. According to the overall sensor parameters, the gain of 200 V/V is required. Thus, matching a resistance (249 Ω) to the AD8221-ARM can provide a gain of 199.4 V/V, which meets the requirement with only one amplification, thereby avoiding the interference signal caused by multiple amplification steps. The chip of the amplifier is connected to the reference voltage (2.5 V) to convert the signals (positive and negative) of the bridge output. The fluctuation and noise of the reference voltage (2.5 V) directly affect the quality of the signal to be processed. Thus, the power supply module is used to provide a standard voltage (5 V), and the high-precision operational amplifier chip is used as the core to design the circuit module that produces a floating reference voltage. High-precision reference voltage (2.5 V) is produced by matching resistance and further processing the data. Finally, the amplified signal is filtered again to remove the interference from the signal amplification process. At this point, the signal is inputted to the processor PIC18F4685 through the front-end processing module to conduct the A/D conversion and the corresponding signal processing. The processed signal is then packed and transferred to the single-leg controller through the CAN bus.

## 4. Force Sensing System Implemented in Control Architecture

### 4.1. Dimensional Transformation of Foot-End 3-DOF Force Sensor 

**Figure 19:**
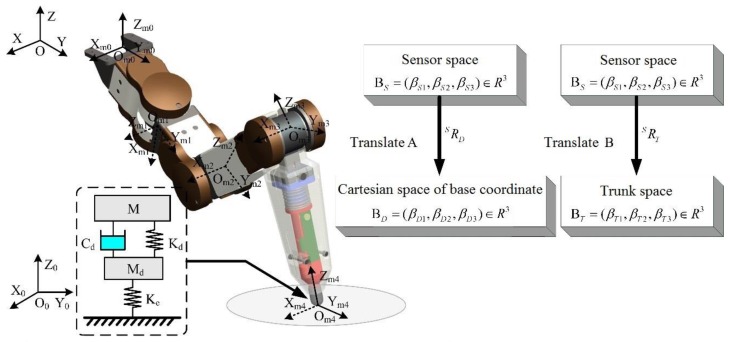
Conversion of sensor space.

When working on the rugged terrain, the postures of the trunk and each leg of the hexapod robot constantly change, thus, the postures of the 3D foot-end force sensor integrated in each tibia of the legs also change. As shown in Figure 19, the measurement results are space force vectors in the sensor based on the foot-end coordinate $\Sigma_{O_{m4}}$, however, the force vector data in different spaces are needed for different requirements. 

For example, the foot-end force in the Cartesian space of the base coordinate is needed when considering the trunk posture, whereas the foot-end force in the trunk coordinate system is needed when considering the foot-end position. Therefore, space conversion is necessary, and the measurement results from the sensor space should be mapped to other spaces. Force vector mapping only requires the rotation of the initial data. The rotation matrix from the foot-end coordinate $\Sigma_{O_{m4}}$ to the trunk coordinate $\Sigma_{O}$ is:

(15) ROm4o=ROm0o·ROm1om0·ROm2om1·ROm3om2·ROm4om3.

Equation (17) yields:

(16) TOmiom(i−1)=[cosθmi−sinθmicosαmisinθmisinαmiamicosθmisinθmicosθmicosαmi−cosθmisinαmiamisinθmi0sinαmicosαmidmi0001]

When $a_{mi}=0$ and $d_{mi}=0$, the rotation matrix can be obtained as follows:

(17) ROmiom(i−1)=[cosθmi−sinθmicosαmisinθmisinαmisinθmicosθmicosαmi−cosθmisinαmi0sinαmicosαmi]

where i≥1. According to Equation (19):

(18) TOm0o=Trans(xom0o,yom0o,zom0o)·Rot(z,βm01)·Rot(x,βm03).

The corresponding rotation matrix is:

(19) ROm0o=Rot(z,βm01)·Rot(x,βm03).

The rotation matrix from the foot-end coordinate $\Sigma_{O_{m4}}$ to the base coordinate $\Sigma_{O}$ is:

(20) ROm4o0=ROo0·ROm4O,

where ROo0=Rot(Z,βr1)·Rot(Y,βr2)·Rot(X,βr3), and $\beta_{r1}$, $\beta_{r2}$, $\beta_{r3}$ correspond to the yaw, pith, and roll angle, respectively, which can be can be directly measured by the position sensor. The force vector $\vec{F}$ map to the trunk space $B_{r}$ and the Cartesian space of the base coordinate $B_{D}$ are $\vec{F_{r}}$ and $\vec{F_{D}}$, which can be expressed as follows:

(21) Fr→=ROm4O·F→,FD→=ROm4O0·F→.

### 4.2. Control Architecture of Hexapod Robot Motion Controller 

**Figure 20:**
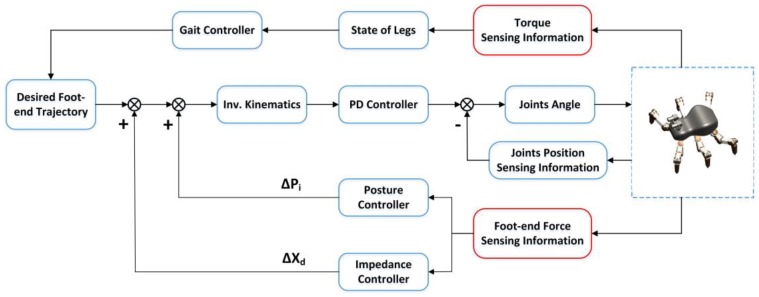
Control architecture of the hexapod robot motion controller.

The force sensing system designed in this paper is not only used to obtain the collision and contact information of the foot-end and the rugged terrain, but also realize the active force control by providing the three-dimensional force sensing information of the foot-end, so as to improve the walking stability of the hexapod robot on unstructured terrains. The control system of the robot is shown in Figure 20. 

The joint torque information is used to judge the contact between the foot end and the ground so as to judge the state of the leg, and provide the leg state information to the gait control to realize the coordinated movement of each leg and to plan the trajectory of each foot. When the robot walks on a rugged terrain, the terrain change makes the robot pose change and this and the collision between the foot and the unknown terrain are the two main factors of walking instability. In this paper, the force sensing system provides foot-end force information to the pose controller and impedance controller, through the active foot-end force control to adjust the posture and improve the stability of the foot-ground force interaction. Finally, the posture control and the impedance controller output the foot-end position compensation Pp and Pi to correct the foot-end trajectory. Using the modified foot trajectory and inverse kinematics and the PD controller we can accomplish precise control of the joint position.

#### 4.2.1. Posture Controller Model

According to the ZMP principle about the constraints of quasi−static motion, when the gravity center projection of the hexapod robot is in the horizontal plane formed by the polygon of the foot-fall point, the robot is in a stable state. Moreover, when the robot is in a very small stable margin state, the support force of each support foot is very different, and the robot is easily flipped from the support foot side. Therefore, in this paper, the foot-force is balancedly distributed to maintain the stability when the robot is walking. Taking the contact point of the *i*-th leg and ground as $P_{i}=[x_{i},y_{i},z_{i}]$, the external force is $F=[F_{x},F_{y},F_{z}]$, the external torque is $M=[M_{x},M_{y},M_{z}]$, the reaction force between foot-end of *i*-th leg with the ground is $f_{i}=[f_{ix},f_{iy},f_{iz}]$, from the vertical component $F_{z}$ of $F=[F_{x},F_{y},F_{z}]$ and the pitch torque $M_{P}$ and rollover torque $M_{R}$ of $M=[M_{x},M_{y},M_{z}]$, the static equilibrium model is obtained as follows:

(22) $$\left\{ \begin{array}{l} \sum_{i=1}^{n}f_{iz}=F_{z}+mg\\ \sum_{i=1}^{n}(x_{i}-x_{G})f_{iz}=M_{P}\\ \sum_{i=1}^{n}(y_{i}-y_{G})f_{iz}=M_{R} \end{array}\right.$$

When 1 ≤ *n* ≤ 6, Equation (1) is expressed as a matrix form as follows:

(23) $$A\times F_{iz}=M^{\prime }$$

where:

(24) $$A=\left(\begin{array}{cccc} 1 & 1 & \cdots & 1\\ y_{1}-y_{G} & y_{2}-y_{G} & \cdots & y_{n}-y_{G}\\ x_{1}-x_{G} & x_{2}-x_{G} & \cdots & x_{n}-x_{G} \end{array}\right)$$

(25) $$F_{iz}={\left[\begin{array}{cccc} f_{1z} & f_{2z} & \cdots & f_{nz} \end{array}\right]}^{T}$$

(26) $$M^{\prime }={\left[\begin{array}{ccc} mg+F_{z} & M_{P} & M_{R} \end{array}\right]}^{T}$$

The formula for the foot-end force is as follows:

(27) $$F_{iz}=A^{-}\cdot M^{\prime }$$

Thus, based on the comparison of the vertical components of the foot-ground interaction force, we can get the horizontal position pose compensation, so as to maintain the stability of the walking motion.

#### 4.2.2. Impedance Controller Model

The impedance control method can coordinate the movement of the leg in the free space and the confinement space, reduce the collision impact, and improve the smoothness of the interaction between the foot and the ground in the support state. The interaction between the foot and the ground is equivalent to a spring-damping second-order coupling system, then the target impedance model of the dynamic behavior of the leg can be expressed as follows:

(28) $$\left\{ \begin{array}{l} M_{d}\ddot{E}+B_{d}\dot{E}+K_{d}^{\prime }E=F_{e}\\ K_{d}^{\prime }=K_{d}+K_{e}\\ E=X_{d}-X_{r}\\ F_{e}=F_{d}-F_{r} \end{array}\right.$$

In where, *F_e_* is The difference between the expectation foot-ground interaction force and actual foot-ground interaction force, which is a 3 × 1 vector; *M_d_*, *B_d_*, *K_d_* is the expected inertia of the leg, the expected damping, the expected stiffness respectively, which are 3 × 3 matrix; $K_{d}^{\prime }$ and *K_e_* are the total stiffness of the interaction system, the stiffness of the ground, which are 3 × 3 matrix; *X_d_* and *X_r_* are desired and actual location of the foot-end, are 3 × 1 vector; *E* is the correction amount of the foot-end position, which is a 3 × 1 vector; *F_d_* and *F_r_* are the expected and actual foot-end interaction force, are 3 × 1 vector; When the legs swing in free space, *F_d_* = 0. According to Equations (4)–(17) the dynamic equation can be expressed as:

(29) $$\begin{array}{ll} F_{l}= & D_{l}({\ddot{X}}_{d}+M_{d}^{-1}B_{d}\dot{E}+M_{d}^{-1}K_{d}E+M_{d}^{-1}K_{e}E-M_{d}^{-1}F_{d})\\ & +C_{l}+G_{l}+F_{d} \end{array}$$

where, *F* is the input control force, 3 × 1 vector; and *D_l_*, *C_l_* and *G_l_* are the leg inertia, Coriolis force and centrifugal force and gravity respectively, which are a 3 × 3 matrix. According to the model, the legs of the hexapod robot are based on their own force perception and control system, and the interaction between the foot and the ground is controlled independently and smoothly. The small foot-end trajectory compensation is overlapped with the desired traces, so as to modify the desired trajectory.

## 5. Experimental Evaluation of Hexapod Robot’s Leg Force Sensing System

The basic performance parameters of the leg sensing system are verified in the front section of the sensor design. This experiment aims to evaluate the function and the overall performance of the robot during typical actual movements.

### 5.1. Crawling Experiment

Crawling motion requires robots to have strong movement ability and for robots to adjust the center-of-gravity position and posture simultaneously according to the center-of-gravity position. The reasonable position adjustment not only improves stability, but also balances the load of each leg. The center-of-gravity position cannot be measured directly, thus, posture and coordination gait should be adjusted according to the leg force perception information.

**Figure 21:**
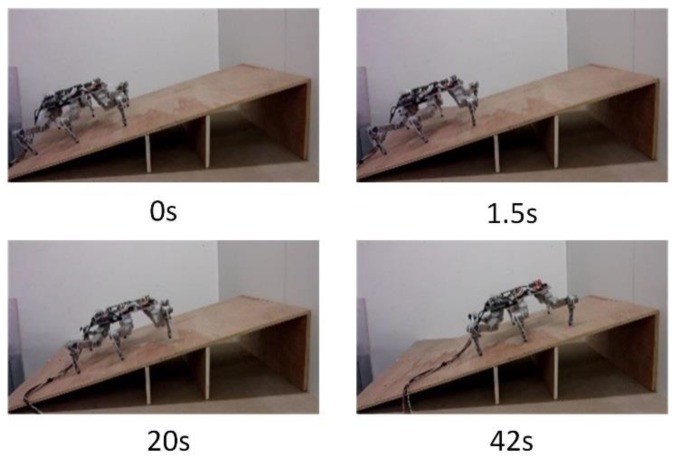
Crawling process of the hexapod robot.

**Figure 22:**
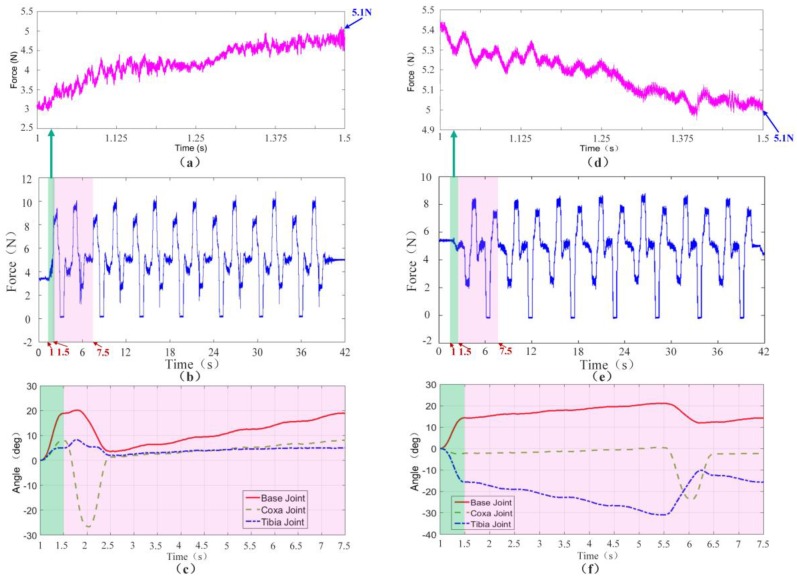
Vertical component of the foot-end force and the joint trajectory of the crawling process: (**a**) Vertical component of the foot-end force of leg L1 during the posture adjustment; (**b**) Vertical component of the foot-end force of leg L1 during the entire crawling process; (**c**) Joint trajectory of leg L1 during the posture adjustment and the first gait cycle; (**d**) Vertical component of the foot-end force of leg L3 during the posture adjustment; (**e**) Vertical component of the foot-end force of leg L1 during the entire crawling process; (**f**) Joint trajectory of leg L3 during the posture adjustment and the first gait cycle.

Figure 21 shows the crawling process of the hexapod robot on a 20° slope. Before starting, the trunk posture is adjusted after comparing the vertical component of the foot-end force of the right foreleg L1 and the right hind leg L3. When the vertical components of foot-end force of legs L1 and L3 are equal, the forward movement of the trunk stops and the crawling motion then starts. As shown in Figure 22b,e, the force sensing system can realize the dynamic detection of the foot-end force during the entire crawling process. The system can clearly reflect the motion state and cycle of the robot through the force detection curve of the entire process. The robot adjusts its position and posture during 1 s to 1.5 s, which is consistent with the angle changes of all robot joints shown in Figure 22c,f. 

**Figure 23:**
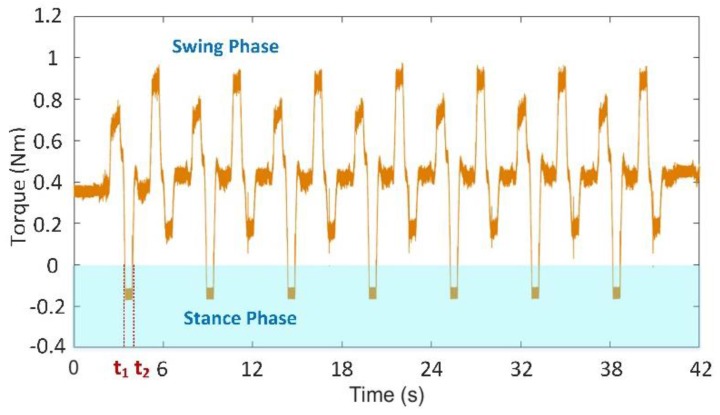
Coxa joint’s moment of leg L1 during the whole crawling process.

During the period from 1.5 s to 7.5 s, the first walking cycle is performed. This force curve period coincides with the gait cycle of gait planning within 6 s. The total weight of the robot is 3.3 kg. The vertical component of the foot-end force in each leg is approximately 5.1 N when the six feet prop up the body together and evenly bear the weight. As shown in Figure 22a,d, position adjustment stops when each vertical component of the foot-end forces finally achieves the same value of 5.1 N. In the actual control process, when the difference of each vertical component of foot-end force in L1 and L3 is smaller than the threshold δ, the position adjustment stops as the initial standard posture due to the non-absolute symmetrical posture and existing uncertain disturbance factors in the quasi-static motion. This result accords with the desired value and verifies that the dynamic measurement of the force sensing system meets the requirements of posture adjustment. The entire climbing process reveals that the robot legs coordinately switch between the support and swing phases and that the motion state is stable. This result shows that the force sensing system can accurately detect the collision and separation between the foot end and the terrain. The motion of the leg can be coordinated based on this information. Figure 23 shows that the value of the joint torque sensor is negative during the swing process due to the weight of the leg. Thus, based on the changes of the state of the leg through the critical point, the leg is in the swing phase when the joint moment is less than the zero point; by contrast, the leg is in the support phase when the joint moment is larger than the zero point. Time t1 switches from the swing phase to the support phase, whereas t2 switches from the support phase to the swing phase. The curve variation period in the climbing process is 6 s, which is consistent with the period of gait planning. Therefore, the force sensing system can accurately perceive the state changes of the leg.

### 5.2. Walking Experiment of Unstructured Rugged Terrain

The ultimate goal of the hexapod robot is to realize autonomous and stable walking on unstructured terrains. The complexity of the terrain needs the robot to feed back the interaction force between the foot end and terrain in a timely and accurate manner, thereby adjusting independently according to the motion and force control models. In view of the motion adjustment strategy of tiger beetles, when a tiger beetle is running on a complex terrain, it adjusts its motion based on the interaction force rather than visual information. Thus, the leg force sensing system of robots should have good comprehensive performance to walk on an unstructured rough terrain. This study aims to evaluate the interaction–force measurement performance of the leg force sensing system on complex terrain.

**Figure 24:**
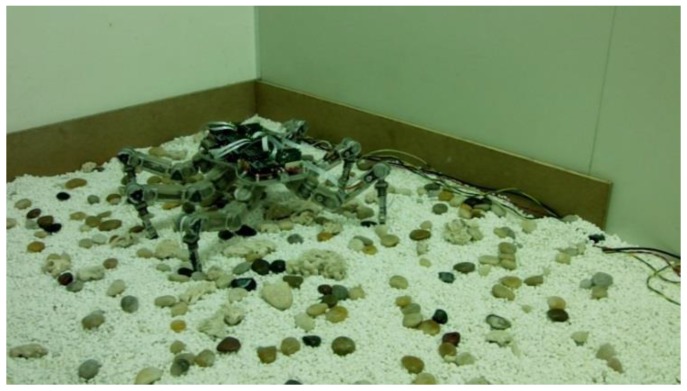
Walking on unstructured rugged terrain.

**Figure 25:**
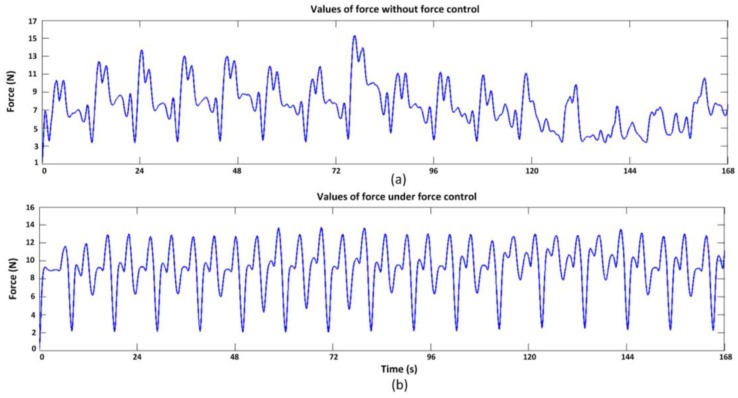
Vertical component of foot-end force when walking on the unstructured complex terrain: (**a**) Measured values of force without force control; (**b**) Measured values of force with foot-end force control.

**Figure 26:**
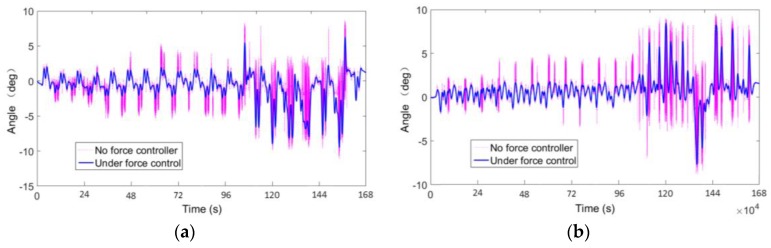
Variation of trunk angle: (**a**) Rolling angle of trunk; (**b**) Pitching angle of trunk.

Figure 24 shows the hexapod robot walking on the terrain, where uneven-sized stones are laid randomly. When walking on this type of terrain, the robot will encounter some complex accidental situations, such as obstacles and ditches, foot sliding, and unstable contacts, which all ask stringent requirements to the dynamic measurement performance and overload protection of the sensor. Figure 25a,b show that the foot-end force control method based on the force-sensing information can effectively reduce the fluctuation of the foot-end force. The force measurement value reflects the gait cycle of the robot and the changes of interaction force with the terrain. Thus, the leg force sensing system designed in this study can effectively measure the interaction force and sense the impact force when the hexapod robot walks on unstructured rugged terrain. The system can still obtain stable measurements even without force control and when the physical interaction is drastic. As shown in Figure 26, the force control method based on force sensing information can effectively reduce the rolling and pitch angles of the robot trunk. 

The deviation value of the pitch angle from the equilibrium position decreases from 3.1 to 2.3, and the value of the rolling angle decreases from 3.4 to 2.5. Moreover, the changes of the foot-end force are smooth, steady, and consistent with the periodic gait variations (Figure 25). Therefore, the leg force sensing system can effectively perceive and measure the interaction force between the leg and terrain. Furthermore, the force controller based on the measured value of this system can effectively improve the stability of the hexapod robot when walking on unstructured complex terrains. Not only can it obtain foot contact state with the ground through single force perception information, or achieve active force control in a single direction, but also adapt to changes of interaction force in complex terrains and accidental irregularities like sliding. In this section, we describe the comparison of robot walking stability in two aspects, one of them obtains foot contact with the ground state through a single force sensing information, another is based on multi−force sensing information for foot−force active control. Thus a comprehensive assessment of the force perception system is revealed.

## 6. Conclusions

In this study, the design and fabrication details of a leg force-sensing system is proposed. Combined with force control methods, this system can help robots recognize and adapt to an unstructured terrain. Torque sensors in the coxa joint and femoral joint, a 3-DOF foot-end force sensor in the tibia part and the force information processing module are designed. Performance parameters of all the sensors refer to the characteristics of the hexapod proposed in this paper, and they are verified by simulation and calibration experiments. The force sensing systems of all six legs are same because of the modularized leg design, and force sensing information combined with force control methods are essential parts of the motion control architecture applied to control the interactive forces between legs and terrain. Finally, the results of the experimental evaluation reveal that the hexapod robot can improve its walking stability in unstructured environments with the aid of the proposed leg force-sensing system. Our future work will focus on combining the force sensing information with posture sensing information to further improve the walking stability on severe rough terrains.

## Figures and Tables

**Table 1:** Design objectives of the sensor parameters.

Unit Name	Range (Nm/N)	Discrimination (Nm/N)	Length (m)	Width (m)	Height (m)
Joint torque	0.002~2	0.002	≤0.026	≤0.026	≤0.01
3-DOF force on foot end	0.003~30	0.003	≤0.09	≤0.18	≤0.18
Processing module	\	\	≤0.07	≤0.35	≤0.32

**Table 2:** Performance parameters of the joint torque sensor.

Name Unit	Range (Nm)	Discrimination (mNm)	Degree of Nonlinearity (%FS)	Hysteresis (%FS)	Repeatability (%FS)	Work Bandwidth (Hz)
value	0.002–2	2	0.16	0.31	0.15	0–1575

**Table 3:** Modal analysis results of 3-DOF force sensor.

Order of Frequency	Value of Frequency	Vibration Type
1	2688 Hz	Translation along Y axis
2	2765 Hz	Translation along X axis
3	4566 Hz	Translation along Z axis
4	6756 Hz	Rotating around X axis
5	7012 Hz	Rotating around Y axis
6	10,126 Hz	Rotating around Z axis

**Table 4:** Error evaluation of foot end 3-DOF force sensor.

Error Type	Fz (%FS)	Fx (%FS)	Fy (%FS)
Type I	0.11%	0.21%	0.14%
Type II	0.0015%	0.0043%	0.0025%

**Table 5:** Performance parameters of foot end 3-DOF force sensor.

Name Unit	Range (N)	Discrimination (N)	Degree of Nonlinearity %FS	Hysteresis (%FS)	Repeatability (%FS)	Work Bandwidth (Hz)
Fz	0.03~30	0.03	0.13	0.19	0.08	0~3044
Fx	0.03~30	0.03	0.24	0.42	0.22	0~1837
Fy	0.03~30	0.03	0.15	0.26	0.13	0~1792
